# Neuronal Signaling by Thy-1 in Nanodomains With Specific Ganglioside Composition: Shall We Open the Door to a New Complexity?

**DOI:** 10.3389/fcell.2019.00027

**Published:** 2019-03-07

**Authors:** Katarina Ilic, Benedikt Auer, Kristina Mlinac-Jerkovic, Rodrigo Herrera-Molina

**Affiliations:** ^1^Croatian Institute for Brain Research, School of Medicine, University of Zagreb, Zagreb, Croatia; ^2^Laboratory of Neuronal and Synaptic Signals, Department of Neurochemistry and Molecular Biology, Leibniz Institute for Neurobiology, Magdeburg, Germany; ^3^Centro Integrativo de Biología y Química Aplicada, Universidad Bernardo O’Higgins, Santiago, Chile

**Keywords:** Thy-1, ganglioside, nanodomain, lipid rafts, neuronal signaling

## Abstract

Thy-1 is a small membrane glycoprotein and member of the immunoglobulin superfamily of cell adhesion molecules. It is abundantly expressed in many cell types including neurons and is anchored to the outer membrane leaflet *via* a glycosyl phosphatidylinositol tail. Thy-1 displays a number of interesting properties such as fast lateral diffusion, which allows it to get in and out of membrane nanodomains with different lipid composition. Thy-1 displays a broad expression in different cell types and plays confirmed roles in cell development, adhesion and differentiation. Here, we explored the functions of Thy-1 in neuronal signaling, initiated by extracellular binding of α_V_β_3_ integrin, may strongly dependent on the lipid content of the cell membrane. Also, we assort literature suggesting the association of Thy-1 with specific components of lipid rafts such as sialic acid containing glycosphingolipids, called gangliosides. Furthermore, we argue that Thy-1 positioning in nanodomains may be influenced by gangliosides. We propose that the traditional conception of Thy-1 localization in rafts should be reconsidered and evaluated in detail based on the potential diversity of neuronal nanodomains.

## Introduction

Thy-1 is a small (17–18 kDa), *N*-glycosylated glycosylphosphatidylinositol (GPI)-anchored protein positioned in outer membrane leaflet domains enriched with cholesterol and gangliosides, called lipid rafts [molecular features, expression patterns and cell functions of Thy-1 are reviewed in Herrera-Molina et al. (2013) and in Leyton and Hagood (2014)]. Thy-1 is expressed in several cell types including human thymocytes, hematopoietic stem cells, glioblastoma cells, mesothelium precursor cells, neurons, and some subsets of fibroblasts among others. Depending on the cell type, the functions of Thy-1 include cell development and differentiation as well as regulation of adhesion and morphological changes in the context of cell-cell and cell-matrix contact (Leyton and Hagood, 2014).

The functions of Thy-1 are proposed to be regulated by the binding of endogenous ligands of which certain integrins are the most prominent ones (Herrera-Molina et al., 2013; Leyton and Hagood, 2014). The first ever characterized Thy-1-Integrin interaction is the one involving extracellular binding of astroglial α_V_β_3_ integrin and changes in the lateral diffusion as well as the nanoclustering state of Thy-1 in the neuronal membrane (Leyton et al., 2001; Maldonado et al., 2017). Notably, the engagement of α_V_β_3_ integrin not only results in profound morphological changes and increased migration in the astrocytes (Avalos et al., 2004, 2009; Hermosilla et al., 2008; Henriquez et al., 2011), but also triggers Thy-1-depending intracellular signaling in neurons (Herrera-Molina et al., 2012, 2013). The α_V_β_3_ integrin-triggered Thy-1 clustering has recently been shown to regulate inactivation and exclusion of the non-receptor tyrosine kinase Src from a Thy-1/C-terminal Src kinase (Csk)-binding protein (CBP)/Csk complex, resulting in p190Rho GTPase activation, cofilin and myosin light chain II phosphorylation, and consequently neurite shortening (Maldonado et al., 2017). However, it remains unknown whether these mechanisms initiated by α_V_β_3_ integrin binding to Thy-1 are occurring in lipid rafts. Interestingly, super-resolution-suited fluorescent analogs of GPI-anchored proteins and gangliosides have recently been developed, expanding the toolbox to evaluate the interactions between these raft-associated molecules (Komura et al., 2016; Suzuki et al., 2017). In particular, these new studies have revealed gangliosides as highly dynamic components of rafts able to interact and regulate positioning of GPI-anchored proteins. Here, we briefly review literature demonstrating that Thy-1 is present in lipid rafts and that, in response to extracellular engagement, its mobility decreases in particular subsets of them. Also, we explore evidence showing that interactions between Thy-1 and raft-associated signaling intermediates occur in a delicate equilibrium within a nanoscale and millisecond time range. Finally, we hypothesize that correct Thy-1 signaling depends on the presence of an adequate lipid milieu and that, particular classes of gangliosides could be important for correct positioning and/or signaling functions of Thy-1 in rafts in the plasma membrane of neurons.

## Thy-1-Containing Lipid Rafts: A Technical and Conceptual Overview

Since Simons and Ikonen postulated the existence of functional lipid rafts (Simons and Ikonen, 1997), this area has been extensively studied in order to clarify the characteristics, composition, and functional role of lipid rafts in living cell systems. The original concept of how lipid rafts are organized, which should be acknowledged, has been subjected to revision and drastically changed over the years. Early experiments almost exclusively used cold detergent to extract these membrane domains and thus they were often accepted to be detergent-insoluble plasma membrane domains (Brown and Rose, 1992). Conceptually, they were thought to be patches of differently organized lipids that house specific transmembrane proteins. Later, additional research evolved the concept of lipid rafts from being stable and long-lived membrane patches to fluid and dynamic arrangements of clustered lipids and proteins (Owen et al., 2012). Although many studies have dwelled on whether lipid rafts even exist, it has become clear that lipid rafts exist and they may occupy only a fractional area of the plasma membrane. More recently, the use of super-resolution microscopy techniques applied to live-cell imaging has revealed rafts as actively changing and dynamically reorganizing nanodomains formed by different lipid and protein composition (reviewed in Sezgin, 2017).

Commonly used procedures for the characterization of lipid rafts are biochemical isolation methods based on ultracentrifugation in sucrose gradients and classical immuno-histochemical protocols (Pike, 2009; Williamson et al., 2010; Aureli et al., 2016). The results derived from these studies vary depending on used detergents, temperature, saline composition of buffers, etc. The choice of detergents is the most critical issue when the goal is to study native lipids, for example cholesterol organization or presence of gangliosides in rafts (reviewed in Klotzsch and Schutz, 2013). As today we know, biochemical isolation of rafts using different non-ionic detergents, namely Triton X-100, can produce a number of artifacts, including non-physiological clustering of certain lipids and proteins. Therefore, classically accepted results obtained using this detergent should be reconsidered and critically subjected to a new scrutiny. A possible way out of this problem could be the introduction of other detergents found to be less disruptive to the plasma membrane and more in tune with the composition and solubility properties of lipid rafts (Chamberlain, 2004; Heffer-Lauc et al., 2005, 2007; Williamson et al., 2010; Sonnino and Prinetti, 2013) (see later).

In contrast to the impossibility of fixing gangliosides, fixation procedures typically with *p*-formaldehyde (PFA) keep proteins in the membrane in immuno-histochemical studies. Clear-cut immuno-histochemical experiments concluded that the inclusion of 1% Triton X-100 (similar concentration is used in most raft isolation protocols) in blocking and primary antibody solutions caused a mild redistribution of Thy-1 from PFA-fixed wild-type to Thy-1 KO brain sections slices when they were incubated together in the same well. This is possibly due to extraction of Thy-1 and incorporation of its lipophilic GPI-anchor in detergent micelles as PFA does not completely fix GPI-anchored proteins (Tanaka et al., 2010). Despite the extractive capacity of the detergent, the immunoreactivity of remaining Thy-1 in different wild-type brain areas was grossly preserved after detergent exposure, indicating that most Thy-1 was fixed and resistant to extraction (Heffer-Lauc et al., 2005, 2007). In PFA-fixed neuronal cultures, Thy-1 staining on the cell surface is very well preserved after the use of Triton X-100-enriched solutions (Herrera-Molina et al., 2012, 2013; Maldonado et al., 2017). These studies showed that Thy-1 (just as any other transmembrane protein) resists detergent-mediated extraction most likely thanks to the fixative-induced cross-linking with other membrane proteins in intimate contact within the lipid raft. Nevertheless, PFA-promoted protein crosslinking is by itself an inevitable pitfall which should be controlled and/or complemented by alternative staining procedures. In neuronal cultures for example, live cell staining with monoclonal antibodies and super-resolution microscopy have been used to confirm changes in Thy-1 clustering (Herrera-Molina et al., 2013; Maldonado et al., 2017). Alternatively, new fixatives have been characterized specially for the use of super-resolution microscopy (Richter et al., 2018).

For years, the inability to isolate rafts at physiological temperature prolonged the debate on the existence of these nanodomains in living cells (London and Brown, 2000; Lingwood and Simons, 2007). Temperature and ion concentration have been proven to influence lipid raft isolation. Chen X. et al. (2009), a publication from Morris’s lab, proposed that the problem of obtaining “physiological rafts” is a technical one caused by the disruption of the inner layer of the plasma membrane when in contact with detergents, such as Triton X-100 dissolved in buffers with inappropriate cation composition (Pike et al., 2002; Schuck et al., 2003; Koumanov et al., 2005). To solve this problem, the authors introduced detergent–containing buffers to mimic the intracellular ionic environment which prevented the disruption of the inner layer of the plasma membrane obtained from rodent brains. In addition to the provided biochemical evidence, the stabilization of membrane domains during isolation at 37°C was demonstrated by obtaining small nano-meso scale rafts of < 100 nm in size, as shown using immune-gold labeled antibodies and electron microscopy (Chen X. et al., 2009; Morris et al., 2011). Furthermore, they showed that the use of the new buffer formulation in combination with the detergents Brij98 or Brij96 further optimizes the isolation of brain rafts at physiological temperature (Chen X. et al., 2009; Morris et al., 2011).

Thy-1 is present in domains enriched with fully saturated lipids, which are distinguishable from prion protein PrP-containing rafts with significantly more unsaturated and longer chain lipids (Brugger et al., 2004). Confirming these results, the existence of independent Thy-1- or PrP-containing domains has been observed in brain membrane preparations with preserved inside-out orientation and isolated at physiological temperature (Chen X. et al., 2009; Morris et al., 2011). These observations strongly support the existence of different lipid raft populations, which are easily distinguishable in their composition. Moreover, it has been shown that Thy-1-containing, but not PrP-containing, lipid nanodomains are associated with actin, strengthening the idea of a tight interaction between Thy-1 and cytoskeletal/ cytoplasmic components (Chen X. et al., 2009; Morris et al., 2011). Therefore, biochemical isolations of lipid rafts have not only provided the basis for the gross understanding of the differences in protein composition, but have also given functional meaning to subclasses of lipidic nanodomains. From this literature (and other), it is clear that GPI-anchored proteins like Thy-1, transmembrane proteins, intracellular signaling intermediates, and a variety of lipids may undergo interdependent interactions to form an undetermined number of different types of rafts.

### Mobility and Nanoclustering of Thy-1 in Lipid Rafts

Biochemical assessments to characterize the presence of Thy-1 in certain rafts have been complemented with high-resolution imaging techniques aiming to observe the localization and behavior of the molecule inside and outside of rafts. More than 25 years ago, classical biochemical experimentation and liquid-phase chromatography revealed that Thy-1 forms multimers of 45–50 and 150 kDa in primary neurons and neuron-like PC12 cells (Mahanthappa and Patterson, 1992). Also, a number of reports have used electron microscopy-associated immunogold particles to describe the spontaneous formation of highly compact nanoclusters as small as 20–100 nm, comprising 2–20 molecules of Thy-1 (Brugger et al., 2004; Chen X. et al., 2009; Morris et al., 2011). More recently, it has been demonstrated that cholesterol in the outer leaflet of the plasma membrane allows tight contact between GPI-anchored proteins like Thy-1, CD59, and even GPI-anchored Green Fluorescent Protein, as these molecules have been observed as close as 4-nm apart using homo-FRET or single molecule tracking (SMT) (Sharma et al., 2004; Chen et al., 2006; Chen Y. et al., 2009; Komura et al., 2016; Suzuki et al., 2017). Thus, it has been proposed that cholesterol-associated nanoclusters of these GPI-anchored proteins may be functional units linked to protein complex formation to regulate signal transduction. This idea is supported by accumulated evidence indicating that the miscibility of lipid components in the plasma membrane may allow the coupling of the outer leaflet with the inner leaflet of the bilayer, facilitating the communication of two proteins on opposite sides of the membrane (Kusumi et al., 2004, 2010; Chen et al., 2006; Chen Y. et al., 2009; Suzuki et al., 2007a,b). Considering this scenario, the coincidental clustering of a critical number of Thy-1 molecules with an environment of saturated lipids in the external layer would act as a trigger for the reorganization of inner leaflet rafts.

Rafts are formed by the lateral assembly of cholesterol, phosphatidylcholine, and sphingolipids like gangliosides in the outer layer of cell membranes (Simons and Ikonen, 1997; Quest et al., 2004). Indeed, cholesterol – despite its rigid and bulky tetracyclic structure - is an essential component as it interacts with other lipids to form 5–200 nm patches with limited stability in the time range of milliseconds to minutes (Kusumi et al., 2004, 2010; Honigmann et al., 2014). From this, it is believed that the proper organization and lipid content in rafts can provide the correct environment for the functioning of more than 250 identified transmembrane and GPI-anchored raft-associated proteins in cell membranes from different sources (Santos and Preta, 2018). The plethora of lipid-protein interactions most likely defines the versatility, stability and specific functionality of these microdomains (Skibbens et al., 1989; Sargiacomo et al., 1993; Danielsen and van Deurs, 1995; Dietrich et al., 2001; Silvius, 2003; Hanzal-Bayer and Hancock, 2007). As an example, both assembly and disassembly of lipid rafts facilitates the effective activation of locally concentrated receptors by extracellular ligands as well as the posterior interaction with downstream effectors, adding speed and specificity to the ligand-receptor-encoded initiation of cell signaling (Pereira and Chao, 2007; Suzuki et al., 2007a,b; Lingwood and Simons, 2010; Pryor et al., 2012). Supporting the dynamism of rafts in terms of heterogeneity and short lifetimes, the use of stimulated emission depletion (STED), SMT, foster resonance energy transfer (FRET), and other super-resolution imaging techniques has helped to visualize protein–protein, protein–lipid, and lipid–lipid interactions becoming transiently stabilized and then disassembled in intact plasma membranes (Honigmann et al., 2014).

Changes in the aggregation state of Thy-1 and other GPI-anchored proteins induced by extracellular engagement have been observed using fast and super-resolution imaging techniques. Additionally, both important technical and conceptual advances have been made in the understanding of the physical dimensions ruling the lateral mobility and clustering of GPI-anchored proteins in cholesterol rafts (Kusumi et al., 2004, 2010; Hell, 2007; Honigmann et al., 2014). Using SMT with a 33-ms resolution, Kusumi’s lab has shown that incubation with antibody-coated 40-nm gold particles clusters 3–9 CD59 molecules, which is enough to promote alternating periods of actin dependent temporary immobilization of the molecules with lifetimes of 200-ms up to 8-s (exponential lifetime = 100-ms) in epithelial and fibroblastic cell lines. The arrested CD59 molecules remained in a compartment of 110-nm in diameter (conventional resolution of fluorescent microscopes 250–400-nm), indicating that immobilization of CD59 is accompanied by limited diffusion in nano-rafts (Suzuki et al., 2007a,b). Jakobson’s lab, also using fibroblasts, SMT with a 33-ms resolution, and antibody-coated 40-nm gold particles, described that the clustering of Thy-1 induces immobilization of the molecule during a slightly broader time rage of 300-ms up to 10-s (Chen et al., 2006). Moreover, both labs demonstrated that the arrest and positioning of the Thy-1 and CD59 clusters in lipid rafts strongly depend on cholesterol integrity. Therefore, it is clear that ultra-fast, but also slow transient arrests of GPI-anchored proteins are triggered by extracellular engagement in cholesterol rafts. Nevertheless, the results obtained using these artificial ligands to promote clustering of the GPI-anchored proteins, like Thy-1, could not fully describe the natural responses to endogenous ligands to a necessary degree.

## Extracellular Binding of Glial α_V_β_3_ Integrin Confines Neuronal Thy-1

For decades, an endogenous ligand for Thy-1 remained in the dark. In 2001, α_V_β_3_ integrin expressed by astrocytes was identified as a receptor for Thy-1 (Leyton et al., 2001). Leyton’s lab has characterized in detail the direct binding between α_V_β_3_ integrin and the RGD-like sequence (RLD, positions 35–37 accession number AAA61180.1) of Thy-1 by surface plasmon resonance (Choi et al., 2005; Hermosilla et al., 2008), confocal microscopy (Herrera-Molina et al., 2012), and recently using molecular force spectroscopy (optical tweezers) (Burgos-Bravo et al., 2018). Importantly, the same lab has revealed crucial α_V_β_3_ integrin-dependent and Thy-1-induced signaling events, promoting morphological changes in astroglial cells (Avalos et al., 2004, 2009; Hermosilla et al., 2008; Henriquez et al., 2011; Alvarez et al., 2016; Lagos-Cabré et al., 2017, 2018; Burgos-Bravo et al., 2018). Supporting a paradigm of bidirectional communication between neurons and astrocytes, the astroglial α_V_β_3_ integrin was found to also act as a ligand for neuronal Thy-1 to trigger signaling events and retraction of axons and dendrites in neurons (Herrera-Molina et al., 2012, 2013; Maldonado et al., 2017).

**FIGURE 1:**
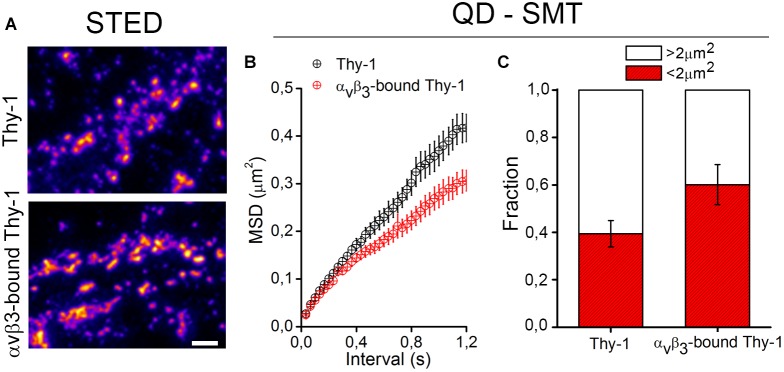
Changes in clustering and confinement of Thy-1 induced upon α_v_β_3_ integrin binding in the neuronal membrane. **(A)** As shown in Maldonado et al. (2017), cultured rat neurons were treated with a soluble form of the α_v_β_3_ integrin, fixed with 4% PFA for 8 min, and stained with a mouse monoclonal anti-Thy-1 antibody (clone OX-7) followed by Atto647N-conjugated secondary antibodies. Then, Thy-1 nanoclusters were visualized using a super-resolution stimulated emission depletion (STED) microscope. **(B,C)** Single molecule tracking (SMT) of Thy-1 molecules attached to quantum dots (QD) is described in Maldonado et al. (2017). Further analysis of mean square displacement (MSD) **(B)** and the total fraction of molecules moving in areas with 2 μm^2^ or more **(C)** confirmed that binding of α_v_β_3_ integrin decreases the lateral mobility and increases the confinement of Thy-1.

The binding of α_V_β_3_ integrin promotes Thy-1 clustering on the neuronal cell surface (Herrera-Molina et al., 2012, 2013; Maldonado et al., 2017). Using super resolution STED microscopy followed by image deconvolution procedures (lateral resolution of 40 nm), single Thy-1 nanoclusters were found as small as 90 nm in diameter (Herrera-Molina et al., 2013; Maldonado et al., 2017). Upon extracellular binding of α_V_β_3_ integrin, Thy-1 clusters with a diameter of 300–400 nm were detected abundantly with extensive aggregation (Figure 1 and Maldonado et al., 2017). Although unitary lifetimes of α_V_β_3_ integrin-bound Thy-1 clusters have not been evaluated yet, a highly dynamic process is expected. Indeed, a single application of α_V_β_3_ integrin was sufficient to reduce the average velocity and displacement area of quantum dot (QD)-labeled Thy-1 molecules, pointing to diminished lateral mobility of α_V_β_3_ integrin-bound Thy-1 clusters (Maldonado et al., 2017). Interestingly, one fraction of Thy-1 molecules (∼60%) was fast (≥5-μm/s), whereas the other one (40%) was comparatively slow (≤5-μm/s) in control neurons. After binding of α_V_β_3_ integrin, a smaller fraction of Thy-1 molecules remained fast (40%) (Figure 1 and Maldonado et al., 2017). Additionally, as α_V_β_3_ integrin binding reduced the mean square displacement (MSD) of Thy-1 molecules (Figure 1), it is possible to speculate that a specific fraction of Thy-1 molecules (20%) is sensitive to the interaction with α_V_β_3_ integrin in neurons. Also, considering the high degree of subcellular compartmentalization of neurons, it is tempting to propose the existence of different subclasses of Thy-1 clusters in dendrites, axons, and/or synapses attending functional specializations of each of these cell compartments.

The evidence points toward a mechanism whereby the clustering of Thy-1 initiates intracellular downstream signals through the single-pass transmembrane adaptor protein CBP (C-terminal Src kinase binding protein). CBP is palmitoylated allowing localization in rafts (Brdicka et al., 1998; Zhang et al., 1998; Chen Y. et al., 2009). CBP plays an obligatory role in the transient arrest of Thy-1 molecules in rafts (Chen Y. et al., 2009) and contains intracellular tyrosine phosphorylation residues that serve as docking sites for Src family kinase (SFK) proteins, including Src and Csk (Wong et al., 2005; Solheim et al., 2008). Both clustering and immobilization of Thy-1 in rafts require SFK activity as demonstrated using QD-associated SMT in fibroblasts (Chen Y. et al., 2009). Moreover, antibody-induced Thy-1 clustering leads to recruitment of SFK to the membrane and modulates the activity of these kinases in a number of experimental settings (Barker et al., 2004; Chen et al., 2006; Yang et al., 2008). In neurons treated with α_V_β_3_ integrin, about 15–20% of Thy-1 nanoclusters have been found to co-localize with CBP as determined using two-channel STED microscopy (Maldonado et al., 2017). Under the same experimental conditions, more CBP co-localized with Csk, which is known to phosphorylate Src at Tyr527 (Chen Y. et al., 2009; Lindquist et al., 2011). Therefore, it was concluded that the binding of α_V_β_3_ integrin to Thy-1 increases the co-localization of clusters of Thy-1, CBP, and Csk in the cell membrane of neurons. Nevertheless, the lipidic nano-environment in which clustering of Thy-1-CBP-Csk took place remains unknown.

## Brief Overview on Neuronal Gangliosides

Gangliosides are sialic acid containing glycosphingolipids, abundantly present in the outer leaflet of the plasma membrane of all cell types (for detailed review of gangliosides see Schnaar et al., 2014). Gangliosides are synthesized in a stepwise manner by sequential addition of monosaccharides on a lipid backbone of ceramide *via* glycosyltransferase activities of different specificity to form oligosaccharide chain. The addition of sialic acid on specific positions in the oligosaccharide chain defines different ganglioside series. Due to their large number and overpowering complexity, gangliosides are still classified by Svennerholm’s nomenclature into groups a, b, and c, depending on the number of sialic acids bound to the internal galactose, and the asialo-group if they have no sialic acid bound to the internal galactose (Svennerholm, 1963, 1980).

Functions of gangliosides include signal transduction, adhesion, cell recognition as well as positioning and function of proteins inside the plasma membrane of neurons (reviewed in detail in Schnaar et al., 2014). The importance of gangliosides for neuronal function has been demonstrated using mutant mice models with disrupted ganglioside synthesis and aberrant ganglioside composition (for example, *B4galnt1*-null mice lack GM2/GD2 synthase expression and thus the four most abundant brain gangliosides (GM1, GD1a, GD1b, and GT1b) are no longer produced). *B4galnt1*-null mice display normal total levels, production, and degradation of cholesterol as well as they do not present any difference in cholesterol turnover compared to wild-type mice (Li et al., 2008). Their phenotype includes axon degeneration, neuropathies, and deficits in reflexes, strength, coordination and posture. Also, male *B4galnt1*-null mice are infertile (Takamiya et al., 1996, 1998; Sheikh et al., 1999; Chiavegatto et al., 2000). At the molecular level, lateral interaction of gangliosides with proteins provides an additional level of regulation of neuronal signaling (Lopez and Schnaar, 2009; Prinetti et al., 2009). Studies have shown that gangliosides can modulate EGF and VEGF receptor sensitivity to their ligands (Bremer et al., 1986; Liu et al., 2006; Mukherjee et al., 2008). Furthermore, endogenous GM1 functions as a specific activator of Trk receptors and is capable of enhancing their activation in response to stimulation with NGF (Suzuki et al., 2004). This effect is most likely due to the enhancement of Trk-associated tyrosine kinase activity elicited by NGF (Mutoh et al., 1995). Therefore, it has been stated that gangliosides are essential regulators of normal neuronal function capable of tuning a number of signaling mechanisms (further argumentation is reviewed in Lopez and Schnaar, 2009; Schnaar et al., 2014).

**FIGURE 2:**
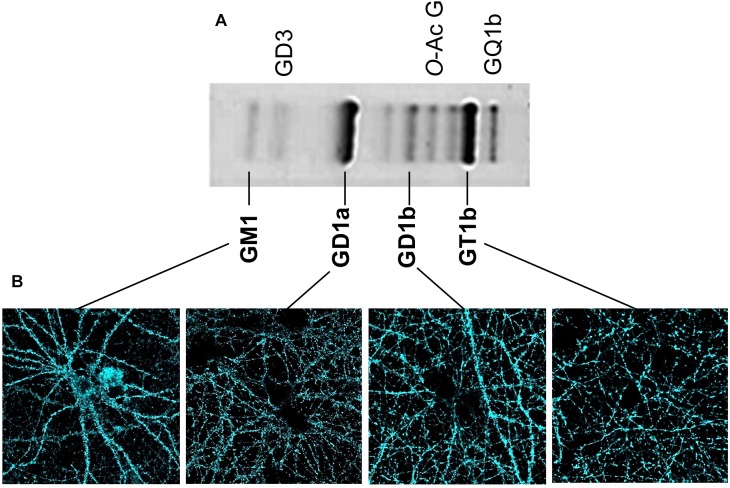
Content of brain gangliosides and visualization of gangliosides in neuronal membrane. **(A)** Separation of the ganglioside types obtained from homogenates of hippocampal cell membranes was performed using HPTLC as described (Svennerholm, 1963). Briefly, gangliosides were extracted from homogenized tissue using a chloroform/methanol/water mix and then purified using a SPECTRA/POR 6 Dialysis Tubing membrane. After drying, samples were spotted on HPTLC plate developed in chloroform/methanol/CaCl2 mix. Gangliosides were detected with a resorcinol-HCl reagent. The identity of each ganglioside type is indicated. **(B)** Confocal microscopy and KO-controlled primary monoclonal antibodies (Schnaar et al., 2002) were used to evaluate independent presence of each of the gangliosides GM1, GD1a, GD1b, and GT1b on the cell surface of living hippocampal neurons. Our procedure to stain living neurons in the absence of fixative and detergents has been described (Herrera-Molina et al., 2012, 2014). Briefly, living rat neurons were directly treated with each of the KO-controlled anti-ganglioside monoclonal antibody diluted in culture media (1:500) for 20 min at 37°C, 5% CO_2_. Then, neurons were carefully washed with culture media, fixed with PFA for 10 min at 37°C, stained with Alexa 488-conjugated secondary antibodies for 1 h, and mounted with Mowiol. All four gangliosides displayed a specific patched signal.

Classical analyses of the expression and distribution of gangliosides have been based on their high extractability with different organic solvents (Svennerholm, 1963). After their extraction, gangliosides have been separated and analyzed using HPTLC (high performance thin layer chromatography) (Figure 2). Additionally, both structural characterization and quantification of the lipid content have been assessed using mass spectrometry (29). These methods have been useful to define the composition and abundance of gangliosides in different tissues and cell types. In the human brain, and very similarly in the rodent brain (Figure 2), GM1, GD1a, GD1b, and GT1b together sum up to 97% of the total ganglioside content. Ganglioside distribution has been studied in brain tissue using specific primary antibodies followed by 3-3′-Diaminobenzidine-based staining similarly as for Thy-1 (Heffer-Lauc et al., 2005, 2007) or other CAMs like Neuroplastin (Mlinac et al., 2012; Herrera-Molina et al., 2017; Ilic et al., 2019). However, special caution is required regarding the detergent used during the procedures as inappropriate conditions produce artifacts as drastic as loss and re-distribution of several ganglioside types. As described by Ronald Schnaar’s lab, the use of some bench detergents, including CHAPS, SDS, and Triton X-100 in PFA-fixed wild-type brain sections, results in a major extraction of gangliosides from their original location (Heffer-Lauc et al., 2005, 2007). The latter effect of detergents was so dramatic that a clear transfer of wild-type gangliosides to the white matter of brain slices of *B4galnt1*-null mice was observed. Authors have optioned to avoid any detergent in ganglioside staining of brain sections. These studies have shown that GM1 is normally concentrated in white matter tracts throughout the adult mice brain, whereas GD1a staining displays a complementary distribution in gray matter. GT1b and GD1b have been found in both gray and white matter (Heffer-Lauc et al., 2005, 2007; Vajn et al., 2013; Schnaar et al., 2014). Unfortunately, in these experimental conditions, uneven antibody diffusion cannot be completely ruled out limiting high-resolution imaging approaches. Lately, we have assessed the visualization and subcellular distribution of the four main brain gangliosides in neurons by combining KO-controlled monoclonal antibodies (Schnaar et al., 2002; Supplementary Figure S1) and high-resolution confocal microscopy. As mentioned before, gangliosides cannot be directly fixed using PFA and they are sensitive to detergent extraction. Therefore, as a first approach, we have used these specific monoclonal antibodies to perform live cell staining either at room temperature or 37°C. Then, one-to-one ganglioside-antibody complexes are fixed with PFA. No detergent is ever used throughout the procedures. Surprisingly, we have obtained a good staining of cell surface located gangliosides (Figure 2). Also, we have visualized the distribution of patches of GM1, GD1a, GD1b, and GT1b throughout soma, dendrites and axons (Figure 2). This promising and simple procedure will be applied to further study these distributions of gangliosides in combination with super-resolution STED microscopy in living neurons. It would be particularly interesting to study the distribution and composition of, what could be, nanodomains differentially enriched with particular gangliosides on the neuronal surface.

## What Can Gangliosides Tell Us About Rafts?

Visualization of the nano-landscape of randomly scattered GM1 patches has been performed with near-field scanning optical microscopy (NSOM). This technique takes advantage of the evanescent field exiting a subwavelength excitation source, therefore being particularly suited for nanoscale optical imaging (≥80 nm of lateral resolution) on intact biological membranes (van Zanten et al., 2010). In particular, organized GM1 nanodomains with a size < 120-nm, separated by an inter-nanodomain distance of approximately 300 nm, were found in the plasma membrane of fibroblasts. Furthermore, this nanodomain organization was not dependent on the temperature, but on the presence of cholesterol and an intact actin-based cytoskeleton (van Zanten et al., 2010). In other studies using antibodies conjugated to gold particles and electron microscopy, either ganglioside GM1 or GM3 were observed forming patches separately and only co-localizing with each other (GM1 and GM3 containing patches) in less than 15% of the cases on the cell surface of fibroblasts (Fujita et al., 2007). Therefore, although composition of lipid rafts can be very divers, their formation and localization seems to be organized throughout the plasma membrane.

Direct visualization of gangliosides and GPI-anchors in living cell membranes has been achieved using chemically synthetized fluorescent analogs and super-resolution STED microscopy (Eggeling et al., 2009; Polyakova et al., 2009; Komura et al., 2016; Suzuki et al., 2017). In 2009, Hell’s lab demonstrated that both Atto647N-conjugated GPI-anchors and GM1 have similar diffusion properties and confinements in rafts (called “trapping”; Eggeling et al., 2009). Notably, the addition of cholesterol-depleting agents similarly reduced the trapping of GPI-anchors and GM1 (Eggeling et al., 2009). Despite recent criticism pointing to insufficient characterization of the fluorescent analogs (Komura et al., 2016; Suzuki et al., 2017), these studies suggest that GPI-anchored proteins and GM1 may share similar lateral diffusion properties in cholesterol rafts. Very recently, a new generation of super-resolution-suited fluorescent analogs has been developed to visualize the relationship between raft-associated GPI-anchored proteins and gangliosides. The authors claimed that the main strength of the new analogs is that they partition in rafts just as the endogenous molecules do (Komura et al., 2016; Suzuki et al., 2017). Furthermore, this method allowed direct observation of positioning and movement of ganglioside and GPI-anchored protein molecules as well as their co-localization without effects of crosslinking. When the raft structure was analyzed by single-molecule imaging, it was determined that ganglioside fluorescent analogs dynamically enter and leave rafts. Inside rafts, ganglioside analogs were immobile for approximately 100 ms, while outside the raft they were constantly moving. The arrest of the ganglioside analogs inside rafts was dependent on actin cytoskeleton and cholesterol integrity (Komura et al., 2016). Additionally, the authors have proposed that cholesterol rafts provide a nano-environment for different proteins, and that gangliosides may have regulatory effects on the recruitment of these proteins. Furthermore, gangliosides could also strengthen interactions between GPI-anchored proteins and other lipids in rafts (Komura et al., 2016; Suzuki et al., 2017).

## Do Gangliosides Influence Clustering/Distribution of Thy-1 in Neurons?

In eukaryotes, gangliosides assemble with other glycosphingolipids and cholesterol to form lipid rafts (Sonnino et al., 2007). It is known that depletion of cholesterol causes deficient clustering of GPI-anchored proteins, including Thy-1 and CD59 (Simons et al., 1999; Sharma et al., 2004; Chen et al., 2006; Chen Y. et al., 2009; Komura et al., 2016; Suzuki et al., 2017), and impairs lipid raft structure (Kabouridis et al., 2000; Buschiazzo et al., 2013). Although, gangliosides have been found to be permissive with the formation of GPI-yellow fluorescent protein clusters in living cell membranes (Crespo et al., 2002), neither deficient nor altered ganglioside content that leads to lipid raft disruption and/or impairs the clustering of GPI-anchored proteins have been studied in detail.

**FIGURE 3:**
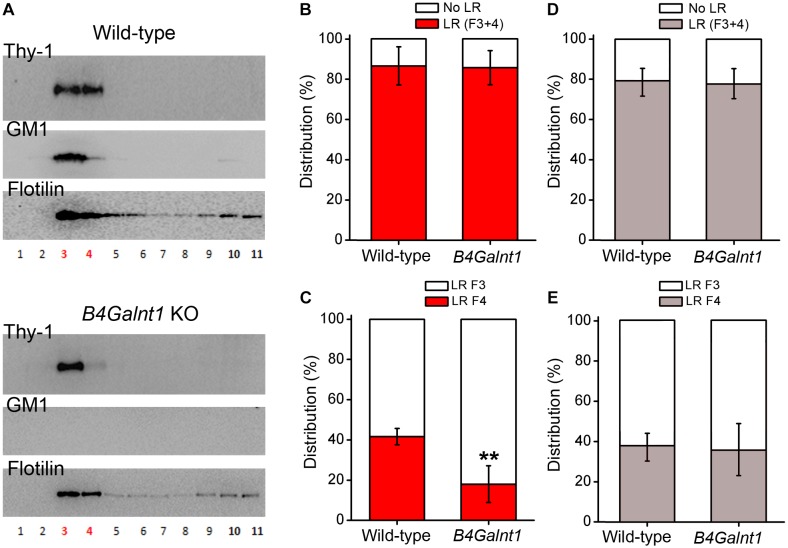
Thy-1 distribution in sucrose density gradients of wild-type and *B4Galnt1-null* brain membranes. **(A)** Representative Western blots of sucrose gradient fractions obtained from total membrane homogenates of wild-type and *B4Galnt1* KO brain cortices as indicated. Lipid rafts isolation is based on published protocols (Persaud-Sawin et al., 2009; Hattersley et al., 2013) with some modifications. After homogenization, nuclear fraction was removed and cell membrane pellet was obtained by centrifugation (30 min, 100,000 × *g*). This pellet was further homogenized in a lysis buffer containing BrijO20 and ultracentrifuged at 140,000 × *g* in a discontinuous sucrose gradient (85% mixed with sample, 35 and 3%). Next day, all fractions were collected for analysis. *B4galnt1*-null mice lacking GM2/GD2 synthase have been previously characterized (Takamiya et al., 1996, 1998; Sheikh et al., 1999; Chiavegatto et al., 2000; Li et al., 2008) and they cannot synthesize any of the four most abundant brain gangliosides GM1, GD1a, GD1b, and GT1b. We confirmed complete absence of GM1 (this Figure) and GT1b (Supplementary Figure S1) in *B4Galnt1* KO brain material using cholera toxin and specific primary antibodies, respectively. Isolation of lipid raft fractions (3 and 4 in red color) was confirmed by detection of flotilin. **(B)** Quantification of accumulative distribution of Thy-1 in bulk membrane fractions (F10 + F11, no lipid rafts: No LR) and in lipid raft fractions (F3 + F4, lipid rafts: LR) of each genotype. **(C)** Quantification of the distribution of Thy-1 in each of the two lipid raft fractions. Distribution of Thy-1 between the raft fractions 3 and 4 seems modified due to the alteration of ganglioside content in *B4Galnt1*-null brain membranes (^∗∗^*P* < 0.01 for fraction 4 comparing genotypes, Mann–Whitney test). **(D)** Quantification of accumulative distribution of flotilin was performed as for Thy-1 in **(B)**. **(E)** Distribution of flotilin between the raft fractions 3 and 4 was performed as for Thy-1 in **(C)**. Data are expressed as mean ± SD of 5 independent gradients.

As mentioned, studies have suggested that gangliosides are important for positioning and clustering of GPI-anchored proteins in cholesterol rafts, rather than being necessary for the raw structuring of the raft itself (Eggeling et al., 2009; Komura et al., 2016; Suzuki et al., 2017). Indeed, authors have reported that positioning of Thy-1 within rafts depends on ganglioside composition as concluded after experiments using cerebellum membrane preparations from wild-type and double mutant mice lacking GM2/GD2 and GD3 synthases (Ohmi et al., 2009). In this study, most Thy-1 immunoreactivity drastically shifted from one to another raft fraction obtained by sucrose gradient centrifugation (Ohmi et al., 2009). Ohmi et al. (2009) concluded that the precise positioning of Thy-1 inside rafts seems to depend on gangliosides. In agreement with Ohmi et al. (2009) we have observed that Thy-1 is present in *B4galnt1*-null lipid rafts (lacking the four main brain gangliosides, see before and Figure 2), but shifted from raft fraction 4 to the lighter fraction 3 (Figure 3). Interestingly, the total content of Thy-1 in *B4galnt1*-null rafts (fraction 3 + 4) was not different to wild-type rafts (also fraction 3 + 4) (Figure 3). Considering that the total content of both cholesterol and sialic acid bound to simpler gangliosides do not differ between *B4galnt1*-null and wild-type mice (Li et al., 2008), our results suggest that altered ganglioside production impaired fine distribution of Thy-1 within *B4galnt1*-null rafts. Supporting this idea, incubation with exogenous GM1 directly and acutely added to the kidney cell line MDCK cells diminished the clustering of the GPI-anchored protein GH-DAF in rafts (Simons et al., 1999). In constructed monolayers of synthetic lipid mixtures with defined lipid composition, the presence of Thy-1 in artificial rafts was found to be reduced when GM1 was added, most likely because GM1 and Thy-1 competed for positioning inside rafts (Dietrich et al., 2001). Thus, it is possible to speculate that exogenously added GM1 formed aggregates reducing Thy-1 mobility in raft-like domains (Marushchak et al., 2007). Although it will be also necessary to proof the potential influence of other lipids as cholesterol, additional available evidence supports the possibility that ganglioside milieu influences Thy-1 location in rafts. Indeed, the literature shows that the distribution of flotilin in raft fractions from neurons, brain tissue, myocites, and erytrocytes is strongly sensitive to cholesterol alterations (Samuel et al., 2001; Kokubo et al., 2003; Jia et al., 2006; Domingues et al., 2010; Sones et al., 2010). These studies consistently show that flotilin distribution reflects and/or reports cholesterol-dependent raft integrity. We shown that the distribution of flotilin is not changed in the *B4galnt1*-null raft fractions with altered ganglioside composition pointing to a rather specific change in raft composition rather than a general modification in the raft integrity (Figure 3).

## Conclusion

For decades, the small GPI-anchored molecule Thy-1 had hidden its charms and remained an orphan in silence. For years, the discovery of Thy-1 as a raft-associated protein served to study these nanoscopic domains. Finally, the development and popularization of super-resolution microscopy techniques allowed to access Thy-1 properties such as lateral mobility, cluster formation, and partition features within the lipidic environment of the cell membrane. The characterization of an endogenous ligand for Thy-1, the α_V_β_3_ integrin, made it possible to reveal detailed mechanisms involved in Thy-1-dependent *cis* signaling in neurons. As experiments show, intracellular signaling emanated from α_V_β_3_ integrin-Thy-1 binding in neurons depends on the initial enrolling of the raft-associated transmembrane transducer CBP and Src kinase to regulate the stability of neuronal cytoskeleton. However, it is still a mystery whether these molecular events are actually occurring in neuronal rafts. The fine-tuning of protein-protein interactions in the outer layer of the cell membrane may be influenced by the lipid environment, in particular by cholesterol and gangliosides, which are two key components of rafts.

**FIGURE 4:**
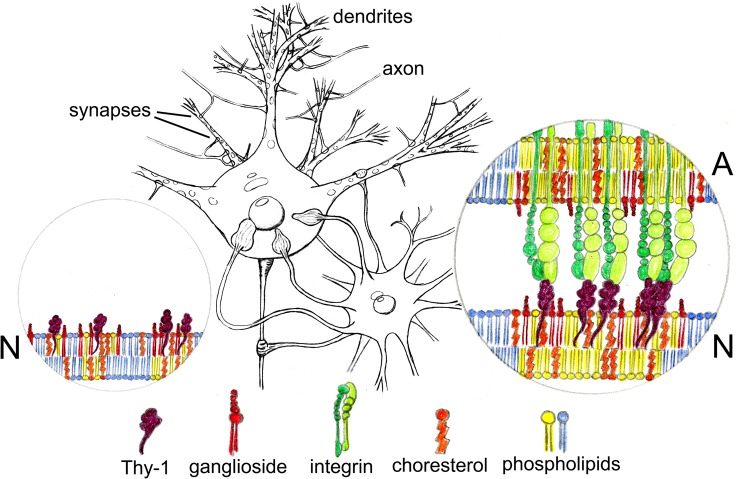
Hypothetical participation of gangliosides in the nanoclustering of neuronal Thy-1 induced by astroglial α_v_β_3_ integrin. Soma, axon, and dendrites of one neuron are contacted by end feet of one astrocyte (**middle drawing**). We propose gangliosides could be important for correct positioning and signaling functions of Thy-1 in rafts in the plasma membrane of neurons. If this turns out to be true, then, gangliosides should influence the diffusion and clustering properties of Thy-1 along the neuronal surface (**N, left circle**) as well as the clustering of Thy-1 molecules induced by α_v_β_3_ integrin expressed by astrocytes (**A, right circle**). α_v_β_3_ integrin-bound Thy-1 might be integrated in nanodomains with particular ganglioside composition to initiate signaling which may differently impact the functioning of each cell compartment.

We propose that the correct ganglioside composition is necessary for distribution, clustering, and function of Thy-1 in neurons (Figure 4). The potential significance of this putative association could be reflected on the capacity of Thy-1 to initiate signaling mechanisms in rafts. In particular, this could be additionally tested by analyzing the α_V_β_3_ integrin-Thy-1-dependent *cis* signaling events that occur at the plasma membrane (Figure 4; Herrera-Molina et al., 2013; Maldonado et al., 2017) in neuronal systems where ganglioside composition is, ideally, acutely modified. Finally, the pieces of the puzzle displayed are waiting to be gathered together into correct assembly.

## Data Availability

All datasets generated for this study are included in the manuscript and/or the Supplementary Files.

## Ethics Statement

Experiments were carried out at Animal Facility of Croatian Institute for Brain Research and were approved by Croatian Ministry of Agriculture, under class number 602-04/14-08/06 and registration number 2158-61-07-14-118.

## Author Contributions

KI contributed the ganglioside isolation, lipid rafts isolation, ganglioside staining, Western blots, and confocal microscopy. BA performed the STED and QD analyses, and graphics. KM-J contributed lipid raft isolation, HPTLC and dot blots. RH-M contributed the work conception, STED and QD experiments, ganglioside staining, confocal microscopy, and wrote manuscript draft. All authors contributed to the final version of the manuscript.

## Conflict of Interest Statement

The authors declare that the research was conducted in the absence of any commercial or financial relationships that could be construed as a potential conflict of interest.

## Supplementary Material

FIGURE S1 Specific detection of the ganglioside GT1b in dot blots and sucrose density gradients using a KO-controlled antibody. **(A)** Representative Western blot analysis of sucrose gradient fractions obtained from total membrane homogenates of wild-type brain cortices as described in Figure 3. GT1b was detected using a mouse monoclonal anti-GT1b previously characterized (Schnaar et al., 2002). Incubation with the primary antibody (1:1000) was followed by a HRP-conjugated anti-mouse secondary antibody (1:5000). The graphics display the quantification of accumulative distribution of GT1b in bulk membrane fractions (F10 + F11, no lipid rafts: No LR) and in lipid raft fractions (F3 + F4, lipid rafts: LR) (left panel) and in each of the two lipid raft fractions (right panel). **(B)** Dot blot analysis served to demonstrate that GT1b is specifically detected in the wild-type but not detected in *B4Galnt1* KO lipid raft fractions.
