# Supplementary figures and images for: Neuronal Signaling by Thy-1 in Nanodomains With Specific Ganglioside Composition: Shall We Open the Door to a New Complexity?
Source: Front Cell Dev Biol. 2019 Mar 7;7:27. doi: 10.3389/fcell.2019.00027 (PMC6416198; doi:10.3389/fcell.2019.00027)

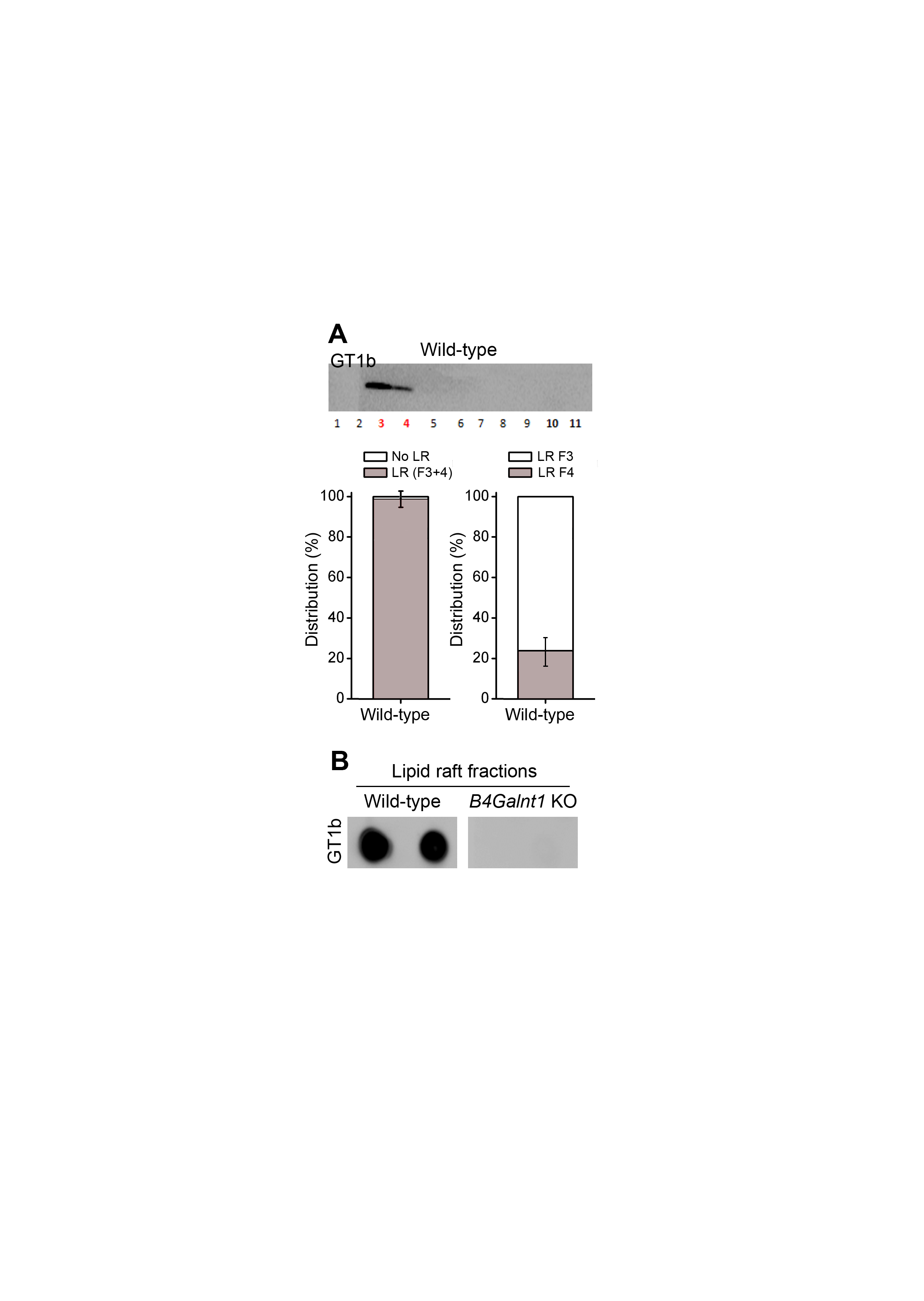

Supplement: FIGURE S1 — Specific detection of the ganglioside GT1b in dot blots and sucrose density gradients using a KO-controlled antibody. (A) Representative Western blot analysis of sucrose gradient fractions obtained from total membrane homogenates of wild-type brain cortices as described in Figure 3. GT1b was detected using a mouse monoclonal anti-GT1b previously characterized (Schnaar et al., 2002). Incubation with the primary antibody (1:1000) was followed by a HRP-conjugated anti-mouse secondary antibody (1:5000). The graphics display the quantification of accumulative distribution of GT1b in bulk membrane fractions (F10 + F11, no lipid rafts: No LR) and in lipid raft fractions (F3 + F4, lipid rafts: LR) (left panel) and in each of the two lipid raft fractions (right panel). (B) Dot blot analysis served to demonstrate that GT1b is specifically detected in the wild-type but not detected in B4Galnt1 KO lipid raft fractions. [file Image_1.tif]
